# RAS–membrane interaction and oligomerization: there is more than meets the eye

**DOI:** 10.1042/BST20253030

**Published:** 2025-08-13

**Authors:** Abraham C. Sianoya, Vijay K. Bhardwaj, Alemayehu A. Gorfe

**Affiliations:** 1 Department of Integrative Biology and Pharmacology, McGovern Medical School, The University of Texas Health Science Center at Houston, Houston, Texas 77030, U.S.A.; 2 Department of Biochemistry and Molecular Biology, College of Medicine, University of the Philippines Manila, Philippines; 3 Molecular and Translational Biology & Therapeutics and Pharmacology Programs, UTHealth MD Anderson Cancer Center Graduate School of Biomedical Sciences, Houston, Texas 77030, U.S.A.

**Keywords:** conformational dynamics, membrane lipids, plasma membrane, RAS protein, signal transduction

## Abstract

Membrane association is fundamental to Rat sarcoma (RAS) function, driving both its physiologic signaling and oncogenic transformation. This review consolidates recent advances in the study of RAS–membrane interactions, emphasizing the molecular mechanisms underlying its membrane engagement and oligomerization. We first discuss the roles of RAS lipid modification and conformational diversity of its intrinsically disordered C-terminus in these processes, and we then examine the debate surrounding RAS dimerization and its potential role in the formation of higher-order oligomers. By integrating emerging insights into these issues, we offer our own perspectives on the driving forces of RAS oligomerization and propose potential new avenues for developing targeted therapies for RAS-driven cancers.

## Introduction

RAS is a peripheral membrane protein composed of three isoforms: HRAS, NRAS, and KRAS (4A and its splice variant 4B). All RAS isoforms act as molecular switches by oscillating between guanosine triphosphate (GTP)-bound active and guanosine diphosphate (GDP)-bound inactive states and mediate signal transduction pathways involved in cell survival and proliferation [1,2]. While RAS possesses a slow intrinsic GTPase activity, its cycling between active and inactive states is facilitated by guanine nucleotide exchange factors (GEFs) and GTPase-activating proteins (GAPs) [3]. Somatic mutations, most frequently occurring at codons 12, 13, or 61, disrupt this cycle by stabilizing RAS in a constitutively active GTP state, thereby driving malignant cell transformation [2,4]. As a result, ~19% of cancer patients harbor a RAS mutation, with KRAS accounting for 75% of RAS-mutant cancers, while NRAS (17%) and HRAS (7%) are associated with a smaller subset [5].

The foundational studies of RAS biology established that the binding of RAS to the cytosolic side of the plasma membrane (PM) is essential for its biological functions (e.g. [6–8]). Progresses in cell biological and biochemical studies of RAS–PM interactions have been reviewed elsewhere (e.g. [9–16]). The current review focuses on recent structural biology, super-resolution imaging, and computational studies that have provided intriguing insights into the molecular details of how RAS proteins interact with and self-assemble on the PM. A more complete picture is emerging on the specific roles of the RAS catalytic domain and hypervariable region (HVR), as well as its interaction partners and PM lipid species, in the membrane engagement and self-assembly of RAS proteins, with important implications for the development of new therapeutic strategies.

### RAS structure, post-translational lipid modification, and trafficking

**Figure 1:**
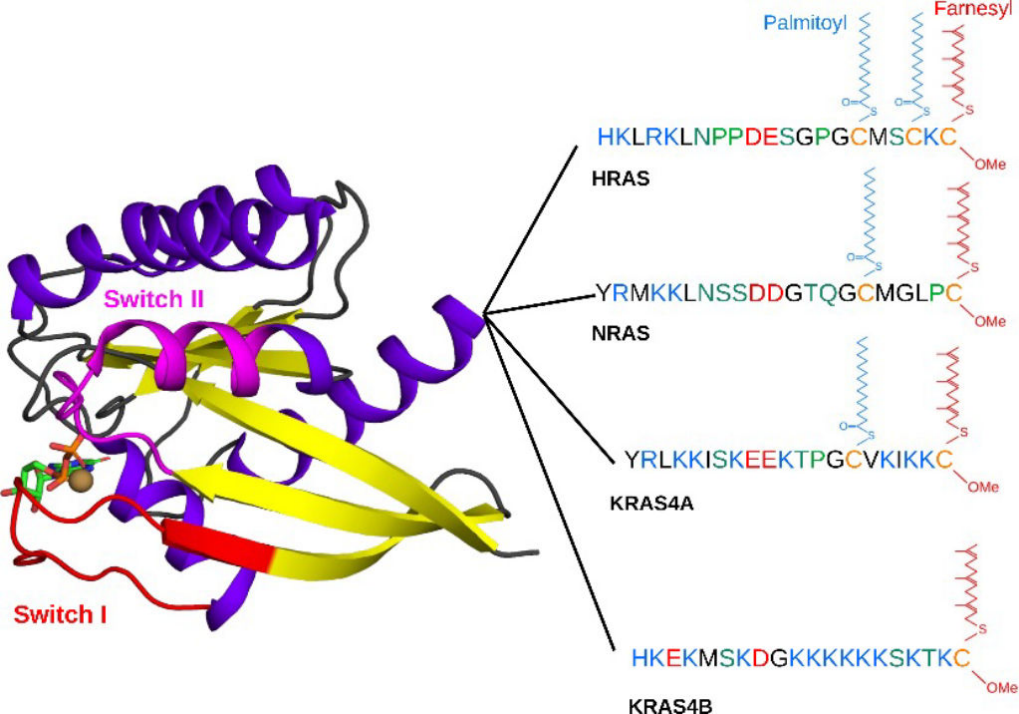
Structure and post-translational modification of RAS proteins. Shown are the conserved 3D structure of the catalytic G-domain and the sequence and lipidation of the hypervariable region (HVR) of NRAS, HRAS, KRAS4A, and KRAS4B. HVR amino acids are color-coded based on chemical properties: blue, basic; red, acidic; green, polar; black, non-polar residues; yellow, lipid-modified cysteine; the nucleotide is shown in atom-colored stick model.

The catalytic G-domain (residues 1–166) of RAS isoforms is highly conserved and shares features typical of other nucleotide-binding proteins, characterized by a six-stranded beta sheet and five helices (Figure 1). The nucleotide (GDP or GTP) binding site and the flexible switch loops SI and SII that change conformation in response to GDP/GTP exchange are located at the G-domain. In addition to GDP/GTP exchange and the resultant conformational changes at the switch loops, RAS function is also regulated by PM binding and unbinding events via the intrinsically disordered HVR (residues 167–185/186) (Figure 1). RAS isoforms significantly differ at the HVR in terms of both its amino acid composition and post-translational lipid modification. Farnesylation (addition of a 15-carbon isoprene lipid to a cysteine residue) is required for PM binding of all RAS proteins, which occurs at the endoplasmic reticulum where a C-terminal CaaX signal (C being Cys, *a* aliphatic, and X any amino acid) is converted to an S-farnesylcysteine carboxymethyl ester through sequential actions of farnesyltransferase, RAS converting enzyme 1, and isoprenylcysteine carboxyl methyltransferase. Insertion of the farnesyl chain into the bilayer hydrophobic core provides the primary driving force for PM binding. In the case of KRAS4B, high-affinity PM binding is further facilitated by the presence of a polybasic domain (PBD) at the HVR, whereas the HVRs of NRAS, KRAS4A, and HRAS need to be further modified by one or two palmitoyl lipids at the Golgi (Figure 1). Fully processed HRAS, NRAS, and KRAS4A are then trafficked to the PM via the exocytic pathway and KRAS4B via recycling endosomes [17–20]. Note that in subsequent sections of this review, we use PBD interchangeably with the phrase ‘polybasic segment’. We use the former to remain consistent with the bulk of existing literature, even though it is somewhat of a misnomer, since the HVR has no domain organization.

### Impact of the HVR sequence and dynamics on RAS membrane binding and lipid sorting

In addition to variations in lipid modification, there is diversity among RAS isoforms in amino acid sequence at the HVR, including in the number and composition of polar, non-polar, and charged amino acids (Figure 1). The positively charged residues at the HVR of the KRAS splice variants, particularly KRAS4B, preferentially interact with acidic lipids such as phosphatidylserine (PS) lipids that are enriched at the inner leaflet of the PM. The palmitoylated HRAS and NRAS, and possibly KRAS4A, are likely to interact with raft-preferring lipid species, including saturated phosphatidylcholine (PC) and cholesterol (see, for example, [21,22]). In addition to inner leaflet PM lipids, it has been shown with electron microscopy (EM) spatial mapping and functional assays that outer leaflet glycosphingolipids, such as GM3 and SM4, modulate KRAS4B’s localization on the PM [23]. A possible mechanism for this was proposed to be cross-bilayer coupling with inner leaflet PS lipids [23].

Molecular dynamics (MD) simulations have shown that the intrinsically disordered HVR of RAS and related small GTPases samples a defined set of conformational ensembles upon membrane binding [24–28]. Sampling of these conformational ensembles was found to depend on the sequence and lipid modification of the HVR, which in turn is correlated with lipid recognition [25–30]. For example, mutating individual residues in the PBD of KRAS4B altered conformational sampling and preference for distinct anionic PM lipid species [29,30]. KRAS4B preferentially sorts asymmetric PS lipid species with one saturated and one unsaturated acyl chains, and the amino acid sequence and prenyl group of the lipid anchor (the C-terminal half of the HVR) are key determinants of this selectivity [29]. Moreover, electroneutral substitutions of lysine (Lys) to arginine (Arg) at the PBD of KRAS4B, RAC1, and RAP1 led to major changes in lipid preferences [29,31,32]. Single-point mutations of individual Lys residues of the PBD to glutamine and the exchange of the farnesyl with geranylgeranyl also resulted in altered lipid specificity [29,31,32]. These intriguing observations led to the conclusion that the prenyl-PBD moiety serves as a combinatorial code for lipid selectivity that goes beyond simple electrostatics (Table 1). In addition to the longer PBD immediately upstream of the farnesyl in KRAS4B, the N-terminus of the HVR of each isoform possesses a short segment of 3–4 Lys, Arg, or histidine residues that may contribute to lipid binding. Of note in this regard is KRAS4A, which contains a second short polybasic segment at the HVR C-terminus between the palmitoyl and farnesyl chains. It was shown that both the N- and C-terminal polybasic segments of the KRAS4A HVR contribute to PM localization, although either one is enough to anchor a prenylated and palmitoylated KRAS4A to the PM [20].

**Table 1:** Summary of studies on the prenyl-PBD code for lipid-binding specificity of RAS superfamily proteins.

GTPase	KRAS-4B	Rac1	RAP1A	RAP1B
Lipid anchor	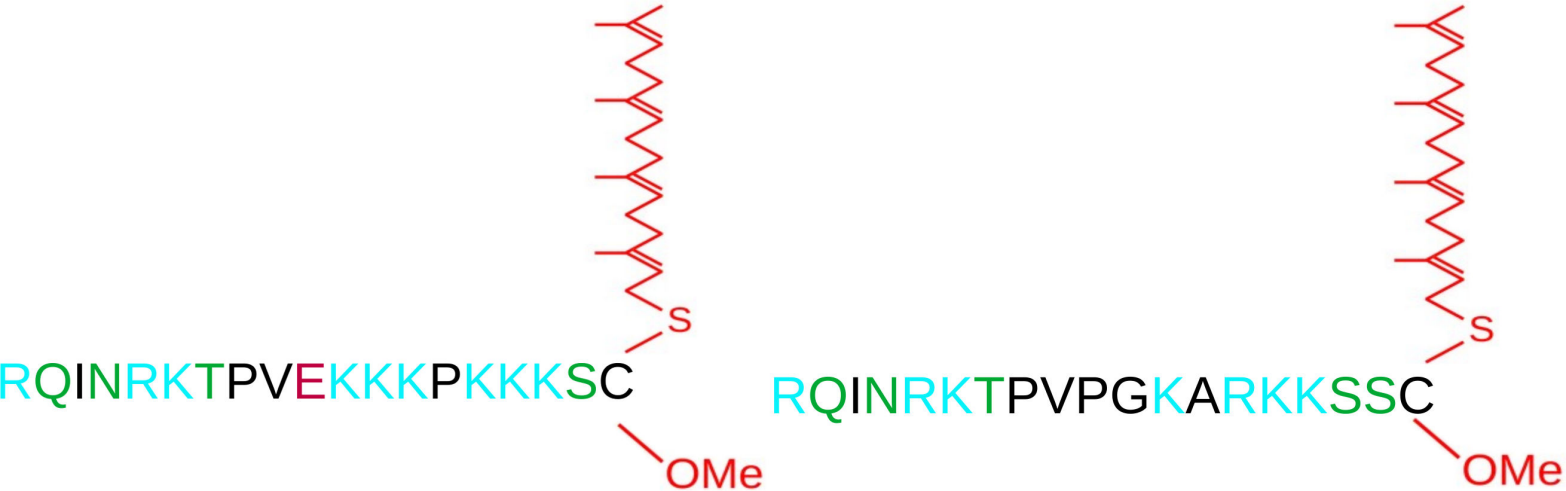	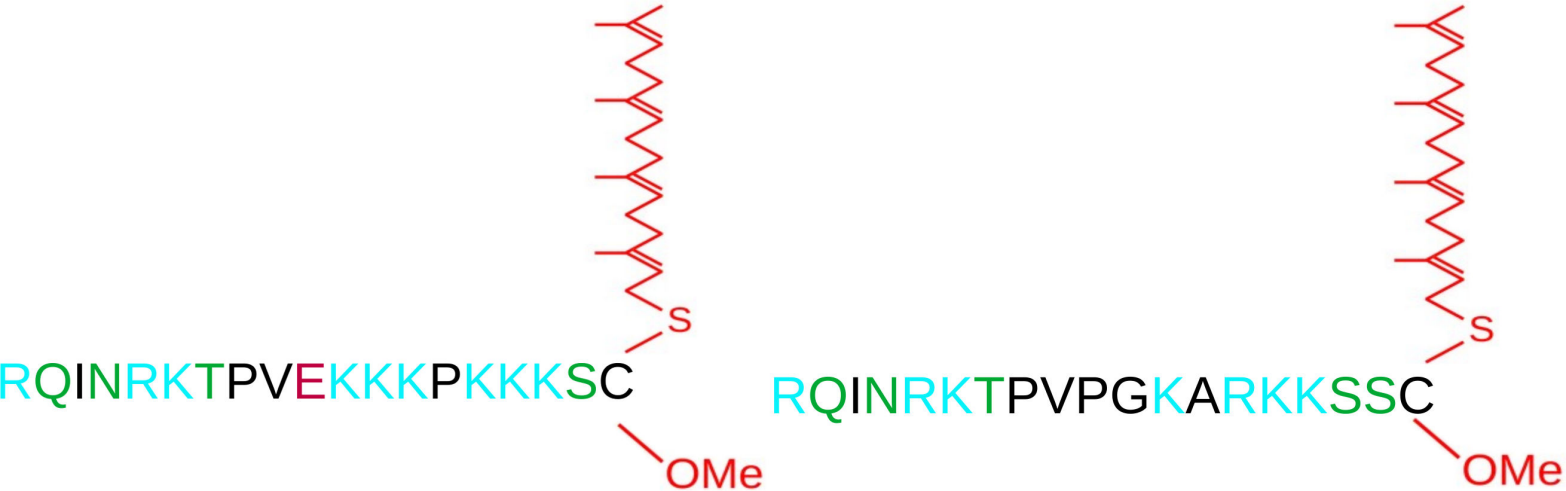	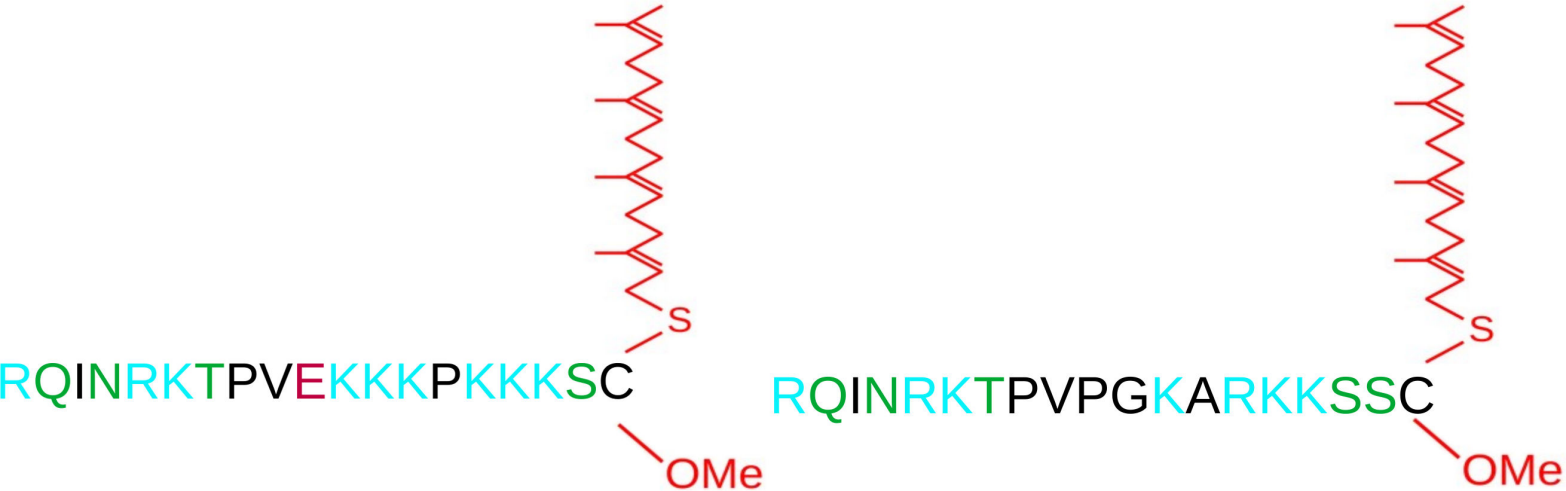	
Cellular function	Cell proliferation, differentiation, survival	Membrane ruffling, cell adhesion, lamellipodium formation, macropinocytosis	Cell migration, micropinocytosis	
Methods	EM-spatial mapping, FLIM-FRET Scanning mutations of PBD residues MD simulations	EM-spatial mapping Scanning mutations of PBD residues	EM-spatial mapping Scanning mutations of PBD residues MD simulations	
Lipids critical for PM binding and clustering	Phosphatidylserine (PS)	Phosphatidic acid (PA) and phosphoinositol (3,4,5)-trisphosphate (PIP3)	PS and PIP3	Cholesterol
Major findings	Clustering with mixed-chain PS species (asymmetric chain length, unsaturated) Prenyl-PBD code for specific lipid binding and effector recruitment	PBD-prenyl/palmitoyl anchor code for lipid-binding specificity; affect micropinocytosis and cell spreading	Spatial segregation of GTP- and GDP-bound Rap1 nanoclusters PM localization of GTP-bound Rap1 is enhanced by effector-dependent interactions Prenyl-PBD codes for lipid binding specificities	
Reference	[29]	[32]	[31]	

Depending on the sequence, the HVR undergoes additional post-translational modifications to further modulate RAS membrane binding or trafficking within the cell. One such example is phosphorylation of KRAS4B at S181 [33,34]. Another example is Lys-ubiquitylation, which was proposed to modulate membrane attachment and cellular distribution of RAS proteins in an activation- and isoform-specific manner [35–37]. Beyond its direct role in membrane binding, the HVR may also mediate interaction with other proteins. A notable example is the interaction of the KRAS4B HVR with phosphodiesterase-δ (PDEδ) for diffusive trafficking within the cell [38–41]. A recent study also showed that PDEδ has a weaker binding affinity for non-palmitoylated KRAS4A due to steric hindrance by the lysine residues upstream of the prenylated cysteine [42]. Moreover, RAS proteins sample multiple membrane orientation states primarily due to the conformational plasticity of the HVR [43–46]. Specifically, KRAS4B was found to oscillate among three dominant orientation states at the membrane, including one in which residues at helices α3 and α4 interact with bilayer lipids and another in which strands β1–3 and α2 helix face the bilayer [21,45,47–49]. These examples highlight the complicated roles of the RAS HVR sequence, lipid modification, and conformational plasticity in PM binding and lipid sorting.

### RAS self-assembly and its modulation by factors other than the HVR

Previous studies have shown that PM-bound RAS proteins form transient, laterally segregated clusters that serve as sites of effector recruitment and activation [50–56]. The HVR has a critical role in this process because, as discussed above, it is required for binding at the PM where clustering occurs. RAS clustering is also modulated by factors that affect the PM structure or dynamics, such as depolarization, curvature, or actions of actin and caveolae [51,56–58]. What is less understood is how RAS self-assembly might be altered by activation status or mutation at the G-domain, membrane lipid composition, or interaction with activators and effectors.

Many lines of evidence suggest that the RAS G-domain and its activation status (GDP- or GTP-loading) have a role in oligomerization. Some of the earliest evidence came from EM analysis showing that the active and inactive forms of NRAS and HRAS do not co-cluster [51,59]. Clustering of KRAS4B-GTP or KRAS4B-GDP is unaffected by cholesterol depletion in cells [59], concordant with atomic force microscopy studies in synthetic membranes showing KRAS4B nanoclusters localizing at the liquid-disordered phase [55]. Despite possible technical limitations, a number of studies using both experimental and computational approaches found that RAS proteins form dimers, including in a nucleotide-dependent manner [60–63]. The best-studied RAS isoform in this regard is KRAS4B. It was proposed earlier that the dominant oligomeric state of KRAS4B and the basic unit to promote functional RAF dimerization is a dimer [60]. A subsequent study using ultraviolet photodissociation mass spectrometry and recombinant KRAS4B found that certain G-domain single-point mutations, such as G12C and G12S, increased KRAS4B dimerization [64]. A more recent single-molecule photobleaching analysis of KRAS4B mutants isolated from Expi293 cells in native lipid nanodiscs found that dimers are more abundant in G12D and Q61H than in WT and that the dimer fraction is higher in KRAS4B G12V pancreatic adenocarcinoma (PDAC) cells than in their WT counterpart [65]. Moreover, native mass spectrometry analysis of KRAS4B reconstituted in PC liposomes found a mixture of monomers and dimers in the GTP state but only monomers in the GDP state [66]. Treatment with a dimer-stabilizing small-molecule ligand (BI-2852) resulted in the loss of the monomer fraction, while treatment with a monobody called NS1, which binds to a potential dimerization interface to prevent oligomerization, favored monomers [66] (Note: BI-2852 and NS1 are discussed further in a later section). Combined with other reports of RAS dimerization (e.g. [67,68]), these findings provide strong evidence for a direct role of the G-domain in oligomer formation.

Considering the likely weak-affinity interactions at the G-domain [62,69–71], additional factors may be required to stabilize RAS dimers and higher-order multimers. There is substantial evidence supporting this notion. In one example, single-molecule tracking experiments found that KRAS4B HVR diffuses almost freely, whereas the full-length protein experienced confinement, suggesting a G-domain-dependent molecular assembly and/or interaction with partner proteins [72]. One of these partner proteins could be the RAS effector RAF, which dimerizes upon binding to RAS [73,74] and modulates RAS-GTP oligomerization [75,76]. While the molecular details of RAS-RAF complexes on the membrane remain incomplete, data from fluorescence correlation spectroscopy and single-molecule tracking on supported bilayers, complemented by MD simulations, led to the conclusion that RAF facilitates KRAS4B dimerization by allosterically exposing a predicted dimerization interface [75]. A combined analysis of data from X-ray, nuclear magnetic resonance (NMR), and MD simulations of KRAS4B in complex with the RAS-binding and cysteine-rich domains of RAF (RAF-RBD-CRD) led to a model whereby both RAS and CRD interact with membrane [12,77]. Similar results were obtained from paramagnetic resonance enhancement NMR experiments of KRAS4B:RAF-RBD-CRD complexes in lipid nanodiscs [78]. A recent study also showed that ARAF sequesters RAS-GTP in biomolecular condensates at the PM [79]. Moreover, the addition of PS or phosphatidic acid (PA), but not phosphatidylethanolamine (PE) or phosphatidylglycerol (PG), in PC liposomes increases the rate of GEF-mediated nucleotide exchange [66], suggesting that RAS-GDP clusters containing specific anionic lipids may enable a more efficient recruitment of GEF. In supported bilayers, there was a delay from PM recruitment to GEF activation, suggesting a complex molecular assembly and condensate formation to activate RAS [80]. Therefore, while the precise roles of effectors and activators remain to be elucidated, it is clear that there is more to RAS oligomerization than meets the eye.

### RAS oligomerization interfaces: the debate continues

**Figure 2:**
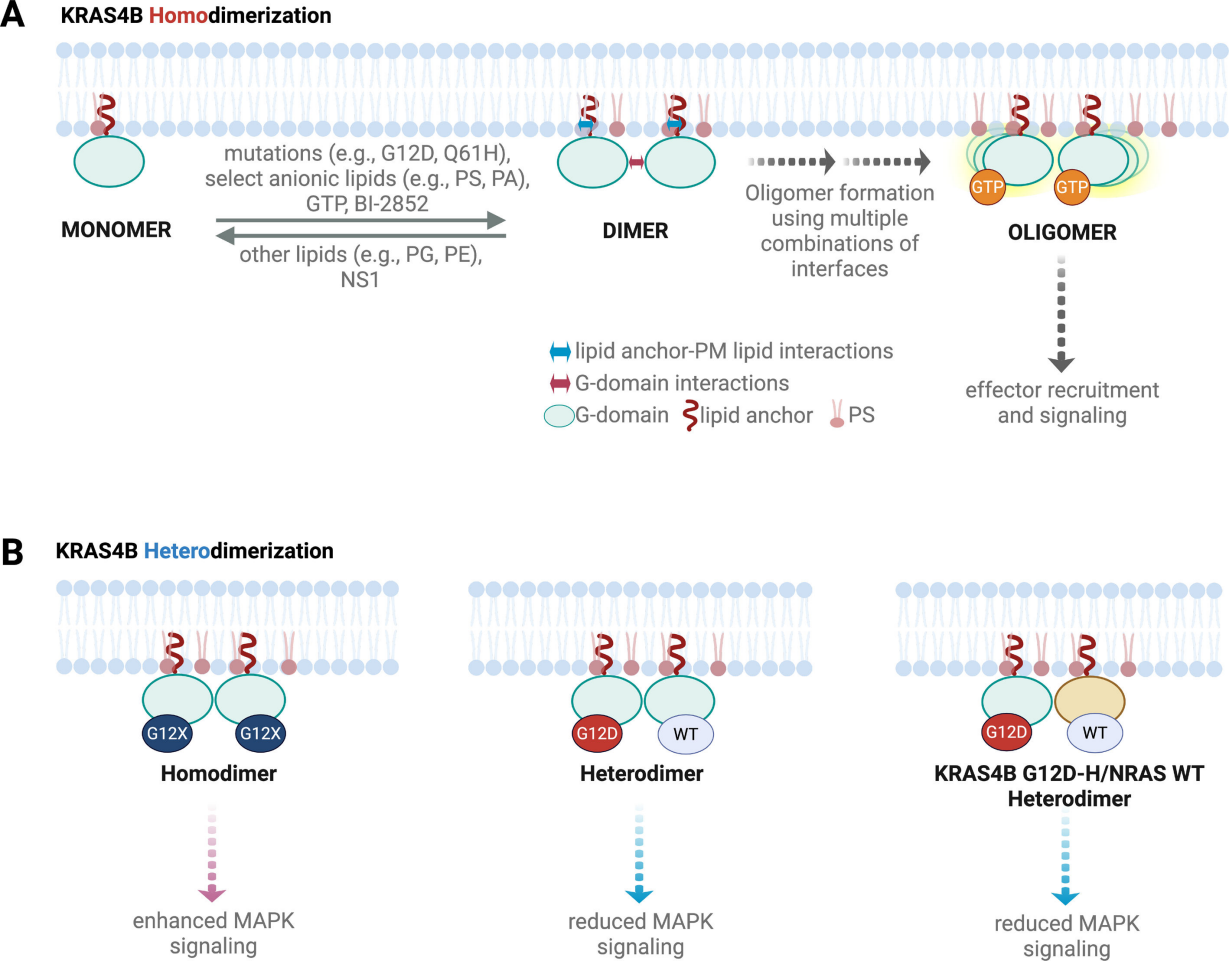
Summary of current evidence for homo- and hetero-dimerization of KRAS4B and their modulation by various factors. (**A**) Examples of oncogenic G-domain KRAS mutations and PM lipid species, as well as GTP and an inhibitor, that favor KRAS4B homodimerization on the one hand, and factors that shift the equilibrium toward the monomer on the other. Note that whether dimer formation is primarily G-domain- or lipid anchor-mediated is still a matter of debate. (**B**) Heterodimerization as a possible explanation of how WT KRAS4B, HRAS and NRAS may modulate the oncogenic function of KRAS4B mutants (see text for details). PS: phosphatidylserine; PA: phosphatidic acid; PG: phosphatidylglycerol; PE: phosphatidylethanolamine; MAPK: mitogen-activated protein kinase. Images created in BioRender.

Despite studies proposing multiple RAS dimerization interfaces [62,69,70,81], many remained unconvinced about the possibility of direct RAS–RAS interaction [82]. Recent findings discussed above and summarized in Figure 2 revived the debate on whether RAS dimers exist as signaling units distinct from nanoclusters or as intermediates of higher-order oligomerization and if dimers arise from G-domain interactions or mediated only by the C-terminal membrane anchor. We argue below that in addition to discrepancies in available information, this debate is a consequence of differences in definition or interpretation (e.g. use of the term ‘dimer’ in the textbook sense) and technical limitations (e.g. limited spatiotemporal resolution of imaging techniques, synthetic versus cell membranes, and fluorescent reporters affecting oligomerization).

**Figure 3:**
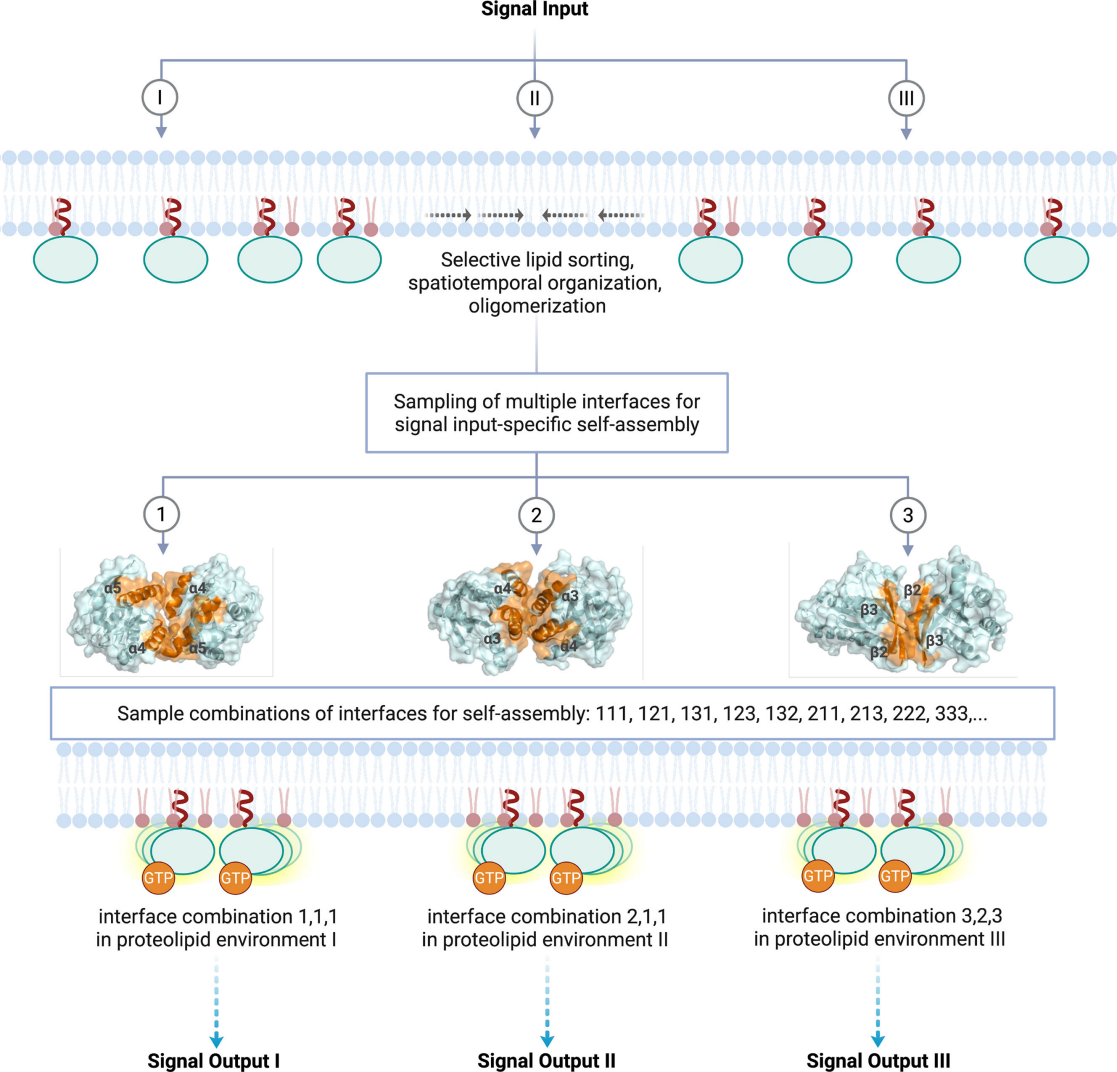
RAS4B samples a combination of interfaces for self-assembly. This proposed model of RAS oligomerization envisages that interfacial promiscuity allows for sampling multiple combinations of interfaces (e.g. interface 1,2,3) to assemble into signal input-specific (e.g. signal I, II, III), topologically and compositionally diverse coexisting multimers that may mediate distinct signal outputs. While this model shares some essential features, such as protein–protein contacts and lipid content, with previously reported models (e.g. refs [9,12]), it emphasizes the multiplicity and lability of interfaces and attempts to shift our view from classical dimers or oligomers to one reminiscent of transient proteolipid condensates. Images created in BioRender.

MD simulations, NMR analyses, and biochemical studies have proposed three major RAS dimer interfaces (reviewed in [9,11–13]): α4-α5, α3-α4, and β2-β3/switch I. We previously showed that the β2-β3/switch I interface is unlikely to have a significant role in RAS function [71] and proposed that monomers may directly assemble into dimers or larger clusters via alternative combinations of the two helical interfaces [83]. The multiplicity of interfaces has been supported by subsequent reports, including from NMR analysis [68,69,84,85]. Taking these together, we now propose that instead of a sequential process of protein–protein interaction followed by lipid sorting, a simultaneous process of protein–protein, protein–lipid, and lipid–lipid interactions underlies RAS oligomerization. In some sense, RAS oligomers may be viewed as reminiscent of molecular condensates characterized by interfacial promiscuity that allows for the formation of coexisting, loosely defined oligomers of different stoichiometries (Figure 3). This notion is consistent with the observations from coarse-grained MD simulation analysis that suggested a potentially large number of interaction surface patches [86], and with the proposition that RAS protomers in close physical proximity may favor effector dimerization without necessarily forming ‘classical’ dimers [82,87]. This concept may also explain observations from fluorescent reporter assays that WT KRAS4B, HRAS, and NRAS impair G12D KRAS4B MAPK signaling, possibly via hetero-dimerization [88] (Figure 2).

As already noted, there is debate about the functional relevance of RAS dimerization and dimer interfaces, particularly regarding the most characterized α4-α5 interface (e.g. [87,89,90]). First, a study in HEK293T and mouse epithelial fibroblast (MEF) cells grown under low-to-starved serum conditions reported that a D154Q mutation at the α4-α5 interface abrogated, presumably through its effect on dimerization, both WT and mutant KRAS4B signaling without affecting intrinsic or GEF- and GAP-mediated activities, RAF binding, or PM localization [89]. A subsequent study in HEK293 cells grown in 10% fetal bovine serum (FBS) failed to observe the dimer-deficient behavior of the same mutant [90], similar to the findings of Whaby et al. [87] that investigated the effect in cells also grown in 10% FBS. Previous single-molecule tracking experiments showed less confinement of KRAS4B in serum-starved cells and increased confinement in media containing 10% FBS, suggesting a possible clustering of GTP-loaded KRAS4B under this condition [72]. A recent effort to reconcile these observations concluded that experimental variables such as growth factors, expression levels, zygosity, and other secondary effectors modulate the outcome of RAS–RAS interactions [91]. It appears that the effect of the D154Q mutation is most evident in cells treated with low concentrations of FBS or fetal calf serum, when small amounts of plasmid are used in transfection experiments, and in the absence of endogenous KRAS4B WT [89,91]. One possible explanation for why the presence of endogenous KRAS4B WT modulates oncogenic functions in exogenously expressed mutant but phenocopies oncogenic behavior in the mut/D154Q setting could be hetero-oligomerization [91]. Considering the lateral segregation of RAS oligomers in an isoform- and nucleotide-dependent manner [55,59,92,93], it would be interesting to test if KRAS4B bearing mutations in dimerization interfaces co-clusters with other KRAS4B mutants. Moreover, the divergent findings so far on the role of dimerization in KRAS4B oncogenic behavior call for approaches that most closely match conditions in cancer cells, such as growth factors and mutant/WT allele frequency, among other factors.

In summary, while it is now clear that membrane binding is required for RAS oligomer formation and that oligomers are held together by weak-affinity interactions, many questions remain open regarding the molecular basis of the process. It appears that neither a protein-centric nor a lipid-centric view is sufficient to describe RAS oligomerization. Instead, we propose a proteolipid hypothesis, whereby weak-affinity protein–protein interactions and lipid-based processes working in concert underlie RAS oligomerization, and that dimers and higher multimers can form simultaneously. In other words, dimers, trimers, or higher oligomers that form through weak-affinity multidentate interactions can coexist with monomers, with the equilibrium among them shifted as needed in a condition-dependent manner. We further propose that different input signals alter RAS–RAS, lipid–RAS, or lipid–lipid interactions differently and thereby change the distribution of oligomers; RAS molecules may utilize different combinations of multiple interfaces for a signal-specific oligomer assembly (Figure 3).

### The RAS G-domain and lipid selectivity

Aside from their role in oligomerization (see previous sections), accumulating evidence suggests that the RAS G-domain and the bound nucleotide contribute to lipid selectivity. Earlier studies have shown that all RAS nanoclusters may contain PS, but only KRAS4B-GTP clusters respond to perturbations in PS content [53,58], and that HRAS-GDP nanoclusters are enriched with phosphatidylinositol 4,5-bisphosphate (PIP2), while those of KRAS4B-GTP have minimal PIP2 [53]. These and other studies (e.g. [72]) strongly suggest activation state-dependent lipid selectivity in cells. The importance of lipid composition for nucleotide-dependent RAS oligomerization has also been demonstrated in synthetic systems. For example, KRAS4B existed as a mixture of fast, intermediate, and slow diffusing species on a supported bilayer made up of a complex mixture of eight lipids but not in a simpler mixture of two lipids, and the confinement of KRAS4B-GTP but not KRAS4B-GDP increases in the presence of BI-2852 [94]. In a more recent study of KRAS4B reconstituted in PC liposomes and analyzed with native mass spectroscopy, a mixture of dimers and monomers was observed for KRAS4B-GTP but only monomers for KRAS4B-GDP [66]. In the former, the dimer fraction increased when PS or PA lipids were added to the PC liposomes, while the equilibrium had shifted toward monomer in the presence of PE or PG [66]. Mutations at the G-domain also alter lipid selectivity. For example, Arora et al. examined the impact of mutating G-domain residues predicted to stabilize certain membrane orientation states of KRAS4B, such as Arg78, finding that these mutations dramatically alter preference for unsaturated PS lipid species [95]. Similarly, some but not all oncogenic KRAS4B mutations altered lipid selectivity, with G12D, G12V, and Q61H KRAS4B co-clustering with mixed-chain PS lipids, while G12C and G13D prefer saturated PS, PIP2, and cholesterol [95].

### Therapeutic approaches targeting RAS membrane binding, dynamics, and oligomerization

Different approaches have been explored over the years to therapeutically exploit membrane targeting of RAS. The earliest efforts focused on RAS farnesylation through the development of farnesyltransferase inhibitors (FTIs) to prevent its localization to the PM [96]. Despite their preclinical success, FTIs failed in clinical trials as monotherapy or in combination with chemotherapies to treat RAS-mutant solid tumors [97,98]. This was attributed to the alternative prenylation (i.e. geranylgeranylation) of KRAS4B and NRAS in the presence of FTIs [99]. Because this alternative prenylation did not affect HRAS [99], FTI treatment in HRAS-driven cancers has shown promise in preclinical models of thyroid cancer, rhabdomyosarcoma, and head and neck squamous cell carcinoma [100–102], and has recently entered multiple clinical trials for different HRAS-mutant cancers [103]. Learning from this experience, a new generation of dual-activity KRAS inhibitors is being developed to target both farnesyltransferase and geranylgeranyltransferase I [104], and covalent modification of C185 in KRAS4B with small molecules revealed a potentially druggable pocket formed by the HVR and α3-α4 [105]. Other approaches to targeting RAS lipidation and trafficking have also been re-examined recently [106].

Therapeutic approaches that aim at dislodging RAS from the PM or disrupting its clustering are also showing promise. A recent example is the identification of a peptide that disrupts HRAS clustering with low micromolar affinity by interfering with the interaction of the scaffolding protein galectin-1 and RAF-RBD [107]. Depletion of PS from the PM by treating cells with fendiline, an acid sphingomyelinase inhibitor, led to KRAS4B PM mislocalization and thereby reduction in oncogenic KRAS4B signaling [108,109]. Moreover, inhibitors of lysophosphatidylcholine acyltransferase, an enzyme that catalyzes the production of unsaturated PS, were found to disrupt KRAS4B clustering and oncogenic function [110]. Other strategies to disrupt PM PS levels include inhibition of its exchange between the PM and ER via oxysterol-related binding proteins ORP5 and ORP8 [111], or silencing phosphatidylinositol phosphatases to reduce PM and total PS levels [112]. Disrupting RAS dimerization may also offer a unique therapeutic opportunity. Along this line, a small protein (NS1) that binds at the α4-α5 interface and designed ankyrin repeat proteins that target the α3-α4 interface inhibit oncogenic RAS signaling, presumably by disrupting oligomer formation [81,113]. Conversely, the small-molecule ligand BI-2852 was found to induce the formation of a non-functional KRAS4B dimer [114]. This suggests that the formation of non-productive RAS dimers, as well as the disruption of functional oligomers, holds therapeutic promises. Finally, it has been shown that RAS adopts multiple distinct orientations on membrane surfaces, including orientation states in which the effector-interacting surface of RAS is occluded by the membrane [43–45,47,49,115]. This raised the question of whether inhibitors that stabilize the occluded orientation state could be found. Two proof-of-concept studies showed that this is possible [116,117]. In one of these studies, a small-molecule ligand was found to simultaneously interact with KRAS4B and the membrane to stabilize an orientation state in which the effector-binding switch loops are occluded [116]. In the other study, a covalent inhibitor (adagrasib) was conjugated with lipids to lock KRAS4B in the inactive GDP-bound form with the effector-binding surface facing the membrane, restricting lateral mobility and disrupting oligomerization [117]. This is just a start, however, and we believe it is inevitable that even more promising therapeutic approaches will emerge as new discoveries on RAS–PM interactions are made.

## Conclusion

RAS proteins are PM-associated molecular switches that engage in a complex relationship with lipids to exert their regulatory function on numerous cellular processes. PM-bound RAS proteins form spatially segregated oligomers in an isoform- and GDP/GTP-dependent manner. The past two decades have seen tremendous progress in further investigating the molecular and, to a lesser degree, structural basis of RAS membrane engagement and oligomerization, both of which are required for RAS signaling. We have discussed the accumulating experimental and computational data on the impact of the G-domain and the HVR on RAS–PM interactions, dynamics, and oligomerization. We have also highlighted how binding to effectors and regulators may contribute to the formation of complex yet highly coordinated molecular assemblies with RAS at the core. However, it remains unclear if higher-order RAS oligomers arise from RAS dimers, and there is no consensus on whether there exists a defined set of protein–protein interaction interfaces underlying dimers or clusters. We advocate for interfacial plasticity to favor diverse coexisting oligomers. We further propose that RAS oligomers are best perceived as somewhat in-between clusters and proteolipid condensates that differ from purely protein- or lipid-based constructs. This view envisages that oligomers are held together by a combination of weak RAS–RAS, lipid–RAS, and lipid–lipid interactions involving diverse, labile, and ‘multi-valent’ interfaces. These same fundamental forces may underlie the self-assembly of other members of prenylated RAS superfamily proteins for which there is evidence of clustering, including RhoA, Rac1, Cdc42, and Rap1A. We anticipate that modern spectroscopic and imaging techniques, including those discussed in this review, will combine with increasingly more powerful modeling and computational methods to provide new insights not only into the distribution of RAS and related G-protein oligomers on membrane surfaces but also into their structure and dynamics. Finally, we have highlighted promising new approaches to therapeutically target RAS–membrane complexes and oligomers, including by modulating RAS–RAS and RAS–membrane interactions as well as by abrogating lipid-dependent RAS clustering.

**Perspectives**

- RAS is an important anti-cancer therapeutic target, and there is a need for new ways of inhibiting its aberrant signaling functions. Promising new approaches are emerging for targeting RAS–membrane complexes and oligomers for cancer therapy, including by modulating RAS–RAS and RAS–membrane interactions.
- There is mounting evidence from experiments and computations on the impact of the RAS G-domain and HVR on its membrane interactions, dynamics, and oligomerization, as well as on the modulation of these processes by effectors and regulators. However, there is no consensus on whether there exists a defined set of protein–protein interaction interfaces underlying RAS dimers or clusters.
- Synthesizing existing information, we propose that RAS oligomers are held together by a combination of weak RAS–RAS, lipid–RAS, and lipid–lipid interactions involving diverse, labile, and ‘multi-valent’ interfaces and may be best perceived as in-between clusters and proteolipid condensates that differ from purely protein- or lipid-based constructs.

## Abbreviations

EM: electron microscopy
FBS: fetal bovine serum
FTIs: farnesyltransferase inhibitors
GAPs: GTPase-activating proteins
GDP: guanosine diphosphate
GEFs: guanine nucleotide exchange factors
GTP: guanosine triphosphate
HVR: hypervariable region
MD: molecular dynamics
NMR: nuclear magnetic resonance
PA: phosphatidic acid
PBD: polybasic domain
PC: phosphatidylcholine
PDEδ: phosphodiesterase-δ
PE: phosphatidylethanolamine
PG: phosphatidylglycerol
PIP2: phosphatidylinositol 4,5-bisphosphate
PM: plasma membrane
PS: phosphatidylserine
